# Generalization of the Right Acute Stroke Prevention Strategies in Reducing in-Hospital Delays

**DOI:** 10.1371/journal.pone.0154972

**Published:** 2016-05-06

**Authors:** Qiang Huang, Hai-qing Song, Xun-ming Ji, Wei-yang Cheng, Juan Feng, Jian Wu, Qing-feng Ma

**Affiliations:** 1 Department of Neurology, Xuanwu Hospital, Capital Medical University, Beijing, China; 2 Department of Neurosurgery, Xuanwu Hospital, Capital Medical University, Beijing, China; 3 Department of neurology, Beijing Tsinghua Changgung Hospital, Medical Center, Tsinghua University, Beijing, China; Massachusetts General Hospital, UNITED STATES

## Abstract

The aim of this study was to reduce the door-to-needle (DTN) time of intravenous thrombolysis (IVT) in acute ischemic stroke (AIS) through a comprehensive, hospital-based implementation strategy. The intervention involved a systemic literature review, identifying barriers to rapid IVT treatment at our hospital, setting target DTN time intervals, and building an evolving model for IVT candidate selection. The rate of non-in-hospital delay (DTN time ≤ 60 min) was set as the primary endpoint. A total of 348 IVT cases were enrolled in the study (202 and 146 in the pre- and post-intervention group, respectively). The median age was 61 years in both groups; 25.2% and 26.7% of patients in the pre- and post-intervention groups, respectively, were female. The post-intervention group had higher rates of dyslipidemia and minor stroke [defined as National Institutes of Health Stroke Scale (NIHSS) ≤ 3]; less frequent atrial fibrillation; higher numbers of current smokers, heavy drinkers, referrals, and multi-model head imaging cases; and lower NIHSS scores and blood sugar level (all P < 0.05). All parameters including DTN, door-to-examination, door-to-imaging, door-to-laboratory, and final-test-to-needle times were improved post-intervention (all P < 0.05), with net reductions of 63, 2, 4, 28, and 23 min, respectively. The rates of DTN time ≤ 60 min and onset-to-needle time ≤ 180 min were significantly improved by the intervention (pre: 9.9% vs. post: 60.3%; P < 0.001 and pre: 23.3% vs. post: 53.4%; P < 0.001, respectively), which was accompanied by an increase in the rate of neurological improvement (pre: 45.5% vs. post: 59.6%; P = 0.010), while there was no change in incidence of mortality or systemic intracranial hemorrhage at discharge (both P > 0.05). These findings indicate that it is possible to achieve a DTN time ≤ 60 min for up to 60% of hospitals in the current Chinese system, and that this logistical change can yield a notable improvement in the outcome of IVT patients.

## Introduction

Intravenous thrombolysis (IVT) is one of the most efficacious treatments for acute ischemic stroke (AIS), for which therapeutic intervention is highly time-sensitive [1,2,3]. Less than half of American [4] and even fewer Chinese [5,6] patients with AIS are treated with recombinant tissue plasminogen activator within the time frame recommended by the American Heart Association and American Stroke Association [door-to-needle (DTN) time ≤ 60 min] [7], which has been validated by the Get With The Guidelines—Stroke program [8]. Facilitating the process of IVT may improve the rate of DTN time ≤ 60 min and consequently, IVT patient prognosis [4,9,10,11,12].

Although strategies for reducing in-hospital delays of IVT administration have been implemented in many Western institutions [4,9,10,11,12] and the median DTN time has been reduced to < 20 min in at least one stroke center [10], this has not been the case in most Chinese hospitals [5,6], which may be attributed to differences between the Chinese healthcare system and those of Western countries [13]. Our previous study showed that the main causes of delays in DTN time were the time taken for decision-making and laboratory tests [6]. In the present historical controlled study, we investigated the efficacy and safety of various strategies for reducing DTN time at a tertiary hospital in China.

## Methods

### Ethics statement

The study protocol was approved by the Ethical Committee of Xuanwu Hospital, and conforms to the principles outlined in the Declaration of Helsinki. Written, informed consent was obtained from all patients.

### Participant eligibility and enrollment

**Fig 1:**
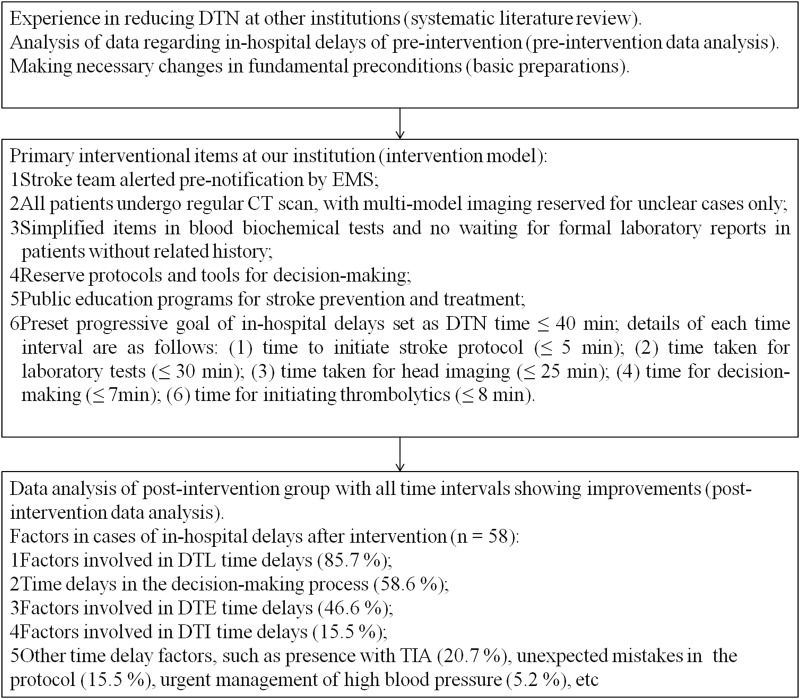
Flow chart of the GRASPs study for reducing in-hospital delays.

The Generalization of the Right Acute Stroke Prevention Strategies (GRASPs) program for reducing in-hospital delay was initiated at the end of 2014. The main items of this program included a systematic literature review, analysis of factors contributing to in-hospital delays at our hospital, and a multilevel implementation strategy that integrated currently available resources and methods for selecting IVT candidates (Fig 1). The following primary data from the literature review were noted: improvements related to emergency medical services [4,9,10,11,14], emergency departments [4,9,10], radiology departments [4,9,10,14,15], and laboratories [10,16]; public education programs [17]; and other organizational changes in the stroke treatment protocol [4,10,12,18,19]. Existing features at our hospital included a neurological unit inside the emergency department, a stroke team and stroke unit on call 24/7, and protocols for emergency endovascular treatment, critical care, and rehabilitation of AIS patients.

AIS patients who visited our hospital through 2015 were recruited as the post-intervention group after the improvement program was implemented in January 2015. The published data for patients enrolled between March 2011 and December 2014 was taken as a historical control (pre-intervention group). And all the AIS cases treated with IVT in 2015 were collected prospectively as the post-intervention group [6]. AIS diagnostic and treatment protocols have been previously described in detail [6], and were slightly modified after the April 2015 update of Chinese guidelines for the care for AIS [20]. In short, the limit of upper age (80 years) and mild stroke severity were removed from exclusions of IVT in the updated guideline [20]. Conferences were held weekly in order to monitor the efficiency of the stroke treatment protocol. The STROBE Statement that should be included in reports of cohort studies was followed (S1 STROBE Checklist).

### Explanatory and outcome variables

Information such as demographic data, stroke severity [as measured by the National Institutes of Health Stroke Scale (NIHSS)], baseline blood pressure and sugar level, medical history of vascular diseases, smoking and drinking status, drug history, and additional factors that were likely to be associated with in-hospital delays [pre-notification from emergency system, urgent blood pressure management, multi-model head imaging, concurrent transient ischemic attack (TIA) or rapidly improving symptoms (RIS)], lesion sites (classified as anterior and posterior circulation according to magnetic resonance imaging results), and admission date and hour were noted [6]. Mild stroke (including RIS) were defined as baseline NIHSS ≤ 3. Outcomes such as neurological improvement (measured as NIHSS), mortality, and systemic intracranial hemorrhage (SICH) at discharge were also recorded. Adverse events related to rapid administration of alteplase, serious bleeding complications for not waiting laboratory results, and erroneous thrombolytics administration for stroke mimics were also recorded. Neurological improvement (NI) at discharge was defined as NIHSS = 0 from a mild baseline (score ≤ 3), improvement by ≥ 4 from a moderate baseline NIHSS (score of 4–9), or improvement by ≥ 8 from a severe baseline NIHSS (score ≥ 10). SICH was defined as any clinical deterioration in NIHSS accompanied by hemorrhage in the 24–36 h after a computed tomography (CT) scan following IVT.

The rate of DTN time ≤ 60 min was set as the primary endpoint, consistent with criteria of the American Stroke Association Target—Stroke initiative [4]. Changes in other parameters, including onset-to-door (OTD), door-to-examination (DTE), door-to-imaging (DTI), door-to-laboratory (DTL), and final-test-to-needle (FTN) times, rates of onset-to-needle (OTN) time ≤ 180 min, and NI at discharge (measured as NIHSS) were set as secondary outcomes. FTN was used to identify the time interval from the last screening test to the needle time of IVT, which included the communication process for IVT decision-making and was equal to 0 when the decision was made before the last test was completed. Mortality and SICH at discharge served as safety indicators.

### Statistical analysis

Statistical analyses were carried out with SPSS v.17.0 software. Continuous variables are presented as median and interquartile range (IQR), and categorical data as percentage (%). The Mann-Whitney U and χ^2^ tests were used to compare related variables between groups, and a two-tailed P-value < 0.05 was considered statistically significant. Multivariable linear regression analysis was performed in order to identify factors contributing to DTN time > 60 min in the post-intervention group, in which variables were selected from the univariate analysis at a significance level ≤ 0.20.

## Results

### Patient characteristics

A total of 348 IVT cases (202 and 146 in the pre- and post-intervention groups, respectively) were recruited for the study. The median age was 61 years (IQR: 52–68 years), and 25.2 and 26.7% of patients were female in the pre- and post-intervention groups, respectively. Demographic and other characteristics of the two groups are shown in Table 1. The post-intervention group showed higher rates of dyslipidemia and minor stroke (defined as NIHSS ≤ 3) and less frequent atrial fibrillation; had a greater number of current smokers, heavy drinkers, referrals, and cases that required multi-model head imaging (all P < 0.05); and lower median NIHSS (score = 4 vs. 9; P < 0.001) and blood sugar level (6.4 vs. 6.9 mmol/l; P = 0.020). The differences in rates among older patients (age ≥ 80 years) did not reach statistical significance (P = 0.878). In 17/146 (11.6%) of IVT cases, pre-notification signals were sent prior to patient admission.

**Table 1:** Baseline characteristics of cases included in this study*.

		Acute ischemic stroke patients treated with IVT		
	Total population (n = 348)	Pre-intervention (n = 202)	Post-intervention (n = 146)	*P*
Age,years,	61 (52–68)	61 (51–69)	61 (53–68)	0.633
Age ≥80 years	9 (2.6)	5 (2.5)	4 (2.7)	0.878
Female	90 (25.9)	51 (25.2)	39 (26.7)	0.758
Medical history				
Hypertension	215 (61.8)	122 (60.4)	93 (63.7)	0.532
Diabetes	106 (30.5)	55 (27.2)	51 (34.9)	0.123
Dyslipidemia	150 (43.1)	75 (37.1)	75 (51.4)	0.008
CHD	56 (16.1)	30 (14.9)	26 (17.8)	0.459
AF	44 (12.6)	32 (15.8)	12 (8.2)	0.035
Prior stroke	77 (22.1)	39 (19.3)	38 (26.0)	0.136
Current smoke	166 (47.7)	111 (55.0)	55 (37.7)	0.001
Heavy drinking	100 (28.7)	69 (34.2)	31 (21.2)	0.009
NIHSS	7 (4–12)	9 (5–12)	4 (3–11)	<0.001
The rate of NIHSS≤3	60 (17.2)	10 (5.0)	50 (34.2)	<0.001
Baseline variables				
SBP(mmHg)	150 (130–165)	150 (130–165)	150 (135–169)	0.383
DBP(mmHg)	85 (80–95)	85 (80–92)	88 (80–96)	0.286
Blood sugar (mmol/l)	6.7 (5.7–8.6)	6.9 (5.8–8.7)	6.4 (5.4–7.8)	0.020
BMI(kg/m^2^)	25.4 (23.5–27.5)	25.0 (23.2–27.4)	25.4 (23.9–27.6)	0.214
Other variables				
Urgent management of BP	40 (11.5)	27 (13.4)	13 (8.9)	0.198
Present as TIA	55 (15.8)	29 (14.4)	26 (18.3)	0.384
Referral	106 (30.5)	70 (34.7)	36 (24.7)	0.046
Transferring with EMS	175 (50.3)	101 (50.0)	74 (50.7)	0.900
Pre-notification	17 (3.2)	0 (0)	17 (11.6)	<0.001
Lesion in AC	282 (81.0)	167 (82.7)	115 (78.8)	0.428
Multi-model imaging	65 (18.7)	59 (29.2)	6 (4.1)	<0.001
Medical insurance	208 (59.8)	113 (55.9)	95 (65.1)	0.087
Working days	246 (70.7)	147 (72.8)	99 (67.8)	0.315
Working hours	191 (54.9)	114 (56.4)	77 (52.7)	0.494

*Unless otherwise stated, continuous and categorical data are presented as median (IQR) and percentage (%), respectively, with P-values calculated using Mann-Whitney U and χ^2^ tests, respectively.

AC, anterior circulation; AF, atrial fibrillation; BMI, body mass index; BP, blood pressure; CHD, coronary heart disease; DBP, diastolic blood pressure; IQR, interquartile range; IVT, intravenous thrombolysis; NIHSS, National Institutes of Health Stroke Scale; RIS, rapidly improving symptoms; SBP, systolic blood pressure; TIA, transient ischemic attack.

All time intervals except OTD were reduced by the intervention (P < 0.05); the net improvement for DTN, DTE, DTI, DTL, and FTN were 63, 2, 4, 28, and 23 min, respectively (Table 2). Improvements were observed in the rates of DTN time ≤ 60 min (pre: 9.9% vs. post: 60.3%; P < 0.001) and OTN time ≤ 180 min (pre: 23.3% vs. post: 53.4%; P < 0.001). Only 33/146 (22.6%) achieved a DTN time ≤ 40 min. The post-intervention group also showed a higher rate of NI (pre: 45.5% vs. post: 59.6%; P = 0.010), but there were no differences in SICH (P = 0.091) or mortality (P = 0.875) at discharge. One case with symptoms that mimicked those of stroke was thrombolyzed, with a final diagnosis of acute clozapine poisoning. There were no serious hemorrhagic complications as a result of not waiting for time-consuming laboratory tests.

**Table 2:** Time intervals and outcomes of cases included in this study*.

	Pre-intervention(n = 202)	Post-intervention(n = 146)	*P*
OTD(min)	110 (67–164)	106 (67–140)	0.510
DTN(min)	116 (93–135)	53 (43–86)	<0.001
The rate of DTN≤60min (%)	20 (9.9)	88 (60.3)	<0.001
OTN(min)	229 (185–270)	173 (130–225)	<0.001
The rate of OTN≤180min (%)	47 (23.3)	78 (53.4)	<0.001
DTE(min)	10 (6–15)	8 (4–13)	<0.001
DTI(min)	28 (15–40)	24 (16–29)	0.002
DTL(min)	84 (67–103)	56 (45–73)	<0.001
FTN(min)	27 (11–45)	4 (0–24)	<0.001
Neurological improvement	92 (45.5)	87 (59.6)	0.010
Mortality	9 (4.5)	6 (4.1)	0.875
SICH	7 (3.5)	11 (7.5)	0.091

*Data are presented as median (IQR) or percentage (%); P-values were calculated using Mann-Whitney U and χ^2^ tests for continuous and categorical variables, respectively.

DTE, door-to-examination time; DTI, door-to-imaging time; DTL, door-to-laboratory time; DTN, door-to-needle time; FTN, final-test-to-needle time; IQR, interquartile range; OTD, onset-to-door time; OTN, onset-to-needle time; SICH, symptomatic intracranial hemorrhage.

There were no statistical differences in baseline characteristics in the post-intervention group, except for the rate of cases with multi-model head imaging between subgroups with or without in-hospital delay (S1 Table). All six cases (4.1%) with multi-model head imaging experienced in-hospital delays, while 17 (11.6%) with pre-notification before admission were treated by IVT without in-hospital delays. Significant improvements in time intervals (DTE, DTI, DTL, and FTN) were also observed in the subgroup without in-hospital delays, although there was no statistical significance in outcome measures (Table 3). Six variables (OTD, DTE, DTI, DTL, FTN, and multi-model imaging) with P ≤ 0.20 in the univariate analysis (S2 Table) were included in multivariate linear regression models, of which FTN (P < 0.001), DTL (P < 0.001), and with multi-model imaging (P = 0.032) were significantly associated with in-hospital delays post-intervention (Table 4). Details of factors influencing in-hospital delays post-intervention are shown in Fig 1.

**Table 3:** Time intervals and outcomes of cases in the post-intervention group*.

	DTN ≤60min (n = 88)	DTN >60min (n = 58)	*P*
OTD (min)	110 (76–157)	100 (58–129)	0.073
DTN (min)	45 (36–52)	98 (79–122)	<0.001
OTN (min)	148 (115–201)	206 (146–245)	<0.001
DTE (min)	6 (3–10)	12 (6–15)	<0.001
DTI (min)	22 (12–28)	27 (20–30)	0.003
DTL (min)	51 (43–61)	72 (55–83)	<0.001
FTN (min)	0 (0–5)	26 (11–37)	<0.001
Neurological improvement	53 (60.2)	34 (58.6)	0.847
Mortality	2 (2.3)	4 (6.9)	0.168
SICH	7 (8.0)	4 (6.9)	0.813

*Data are presented as median (IQR) or percentage (%). P-values for continuous and categorical variables were calculated with the Mann-Whitney U and χ^2^ tests, respectively.

DTE, door-to-examination time; DTI, door-to-imaging time; DTL, door-to-laboratory time; DTN, door-to-needle time; FTN, final-test-to-needle time; IQR, interquartile range; OTD, onset-to-door time; OTN, onset-to-needle time; SICH, symptomatic intracranial hemorrhage.

**Table 4:** Multivariate linear regression analysis of independent variables affecting in-hospital delays post-intervention*.

Variables	Standardized coefficient	*P*
Onset-to-door time	-0.041	0.529
Door-to-evaluation time	-0.006	0.949
Door-to-imaging time	0.091	0.218
Door-to-laboratory time	0.336	<0.001
Final-test-to-needle time	0.480	<0.001
CT perfusion imaging	0.140	0.032
Pre-notification	-0.139	<0.001

*In-hospital delays were defined as DTN times > 60 min.

## Discussion

It is important to design a multi-level implementation strategy for reducing in-hospital delays of IVT administration that fits the specific organization. To our best knowledge, this is the first report that incorporates experiences from more advanced institutions and progressive self-reform in Chinese hospitals. Our study showed that it is possible to achieve a DTN time of ≤ 60 min at as many as 60% of hospitals in the Chinese healthcare system—and thereby improve the short-term prognosis of IVT cases—by implementing organizational changes.

Reducing delays in DTL and FTN times contributed the most to the improvement in DTN time at our hospital. We identified specific factors causing time delays and limiting accessibility to IVT in our earlier studies [6,21], which allowed us to target our improvement efforts. The best practices from other studies [14,22,23] as well as from our own previous work [6] included simplifying as much as possible the items in screening tests (including simplified blood biochemical tests and a single CT scan for head imaging), not waiting for formal laboratory reports for patients without related history, and negotiating with IVT candidates using reserve protocols and tools (e.g., informed outcome diagrams for IVT). As highlighted in the TARGET: STROKE trial protocol [24], implementing organizational changes requires highly coordinated, multilevel, and focused effort. The success of GRASPs was also attributed to the coordinated efforts of all related departments, including but not limited to the neurology and neurosurgery departments. A clear target in the DTN time frame and a prompt data feedback system were invaluable in facilitating cooperation in the IVT candidate selection protocol.

However, there remain shortcomings in the Chinese hospital network as compared to more advanced institutions [9,10,14]. The main problems were difficulty in implementing a round-the-clock point-of-care laboratory test with sufficient staff and other resources, a low rate of pre-notifications from EMS, and delayed activation of the system for fully loaded neurologists and nurses in the emergency department. Less familiarity with stroke symptoms, especially in cases of mild stroke, may have contributed to DTI time delays in our study; to this end, training programs in stroke symptom recognition for emergency physicians, nurses, and radiologists could be helpful [13]. However, even well-designed randomized controlled trials with a fixed implementation strategy may achieve only mild-to-moderate progress in promoting IVT for AIS [25,26]. Modern guidelines with detailed recommendations for reducing IVT time delays, national stroke initiative programs (e.g., the TARGET: STROKE trial [4,24]), and effective monitoring protocols for stroke care are still needed in China.

Recently, programs for reducing in-hospital delays were explored in highly developed hospitals equipped with the modern technologies and skilled staff. Mobile stroke treatment units are now available in Germany and the USA [27,28], which could potentially minimize in-hospital delays for IVT candidates. However, due to the unavailability of specialized equipment and other problems unique to the Chinese healthcare system [13], such units may not be realistic for most of Chinese hospitals at present. On the other hand, pre-hospital factors merit greater attention [29] and may also contribute to the reduction in IVT treatment time delays [18].

Our study had some limitations. Firstly, the experiences of a single hospital may not apply to other institutions. However, the positive results of the GRASPs study provide a basis for self-reform at other hospitals. Moreover, we were unable to draw a firm conclusion such as that of the Target: Stroke initiative [4], since not all endpoints (e.g., mortality and SICH) were improved following the intervention, possibly due to the relatively small sample size.

## Conclusions

Our study demonstrated favorable results in GRASPs for reducing in-hospital delays in IVT treatment. Organizational changes were accompanied by significant improvements in patient outcome.

## Supporting Information

S1 STROBE Checklist STROBE Statement—Checklist of items that should be included in reports of cohort studies. (DOC)

S1 Table Baseline characteristics of cases in the post-intervention group. *Unless otherwise stated, continuous data are presented as median (IQR); P-values were calculated with the Mann-Whitney U and χ^2^ tests for continuous and categorical variables, respectively. AC, anterior circulation; AF, atrial fibrillation; BMI, body mass index; BP, blood pressure; CHD, coronary heart disease; DBP, diastolic blood pressure; EMS, emergency medical service; IQR, interquartile range; IVT, intravenous thrombolysis; NIHSS, National Institutes of Health Stroke Scale; SBP, systolic blood pressure; TIA, transient ischemic attack.
(DOC)

S2 Table Univariate linear regression analysis for identification of independent variables influencing in-hospital delays post-intervention. *In-hospital delay was defined as DTN time > 60 min. AC, anterior circulation; EMS, emergency medical service; NIHSS, National Institutes of Health Stroke Scale; TIA, transient ischemic attack.
(DOC)
