# Supplementary material for: Generalization of the Right Acute Stroke Prevention Strategies in Reducing in-Hospital Delays
Source: PLoS One. 2016 May 6;11(5):e0154972. doi: 10.1371/journal.pone.0154972 (PMC4859531; doi:10.1371/journal.pone.0154972)
Supplement: S1 Table — *Unless otherwise stated, continuous data are presented as median (IQR); P-values were calculated with the Mann-Whitney U and χ2 tests for continuous and categorical variables, respectively. AC, anterior circulation; AF, atrial fibrillation; BMI, body mass index; BP, blood pressure; CHD, coronary heart disease; DBP, diastolic blood pressure; EMS, emergency medical service; IQR, interquartile range; IVT, intravenous thrombolysis; NIHSS, National Institutes of Health Stroke Scale; SBP, systolic blood pressure; TIA, transient ischemic attack. (DOC) [file pone.0154972.s002.doc]

**S1 Table. Baseline characteristics of cases in the post-intervention group** [If not otherwise stated, continuous data were presented as median (IQR) , categorical data were presented as percentage (%), *P* values were calculated using Mann-Whitney U test for continuous variables and Pearson Chi-Square test for categorical variables. IQR indicates interquartile range; IVT, intravenous thrombolysis; NIHSS, National Institutes of Health Stroke Scale; CHD, coronary heart disease; AF, atrial fibrillation; SBP, systolic blood pressure; DBP, diastolic blood pressure; BP, blood pressure; BMI, body mass index; TIA, transient ischemic attack; AC, anterior circulation; EMS, emergency medical service.]

|  | **Total population (n=146）** | **DTN≤60min(n=88)** | **DTN˃60min(n=58)** | ***P*** |
| --- | --- | --- | --- | --- |
| Age,y,  Age ≥80y | 61(53-68)  4(2.7) | 61(54-68)  1(1.1) | 62(52-69)  3(5.2) | 0.978  0.144 |
| Female | 39(26.7) | 25(28.4) | 14(24.1) | 0.568 |
| Medical history |  |  |  |  |
| Hypertension | 93(63.7) | 58(65.9) | 35(60.3) | 0.494 |
| Diabetes | 51(34.9) | 34(38.6) | 17(29.3) | 0.247 |
| Dyslipidemia | 75(51.4) | 42(47.7) | 33(56.9) | 0.278 |
| CHD | 26(17.8) | 15(17.0) | 11(19.0) | 0.767 |
| AF | 12(8.2) | 6(6.8) | 6(10.3) | 0.448 |
| Prior stroke | 38(26.0) | 20(22.7) | 18(31.0) | 0.263 |
| Current smoke | 55(37.7) | 31(35.2) | 24(41.4) | 0.453 |
| Heavy drinking | 31(21.2) | 16(18.2) | 15(25.9) | 0.267 |
| NIHSS | 4(3-11) | 4(3-10) | 4(3-12) | 0.504 |
| The rate of NIHSS≤3 | 50(34.2) | 30(34.1) | 20(34.5) | 0.961 |
| Baseline variables |  |  |  |  |
| SBP(mmHg) | 150(135-169) | 151(130-170) | 150(136-164) | 0.962 |
| DBP(mmHg) | 88(80-96) | 90(80-97) | 87(80-95) | 0.745 |
| Blood sugar (mmol/l) | 6.4(5.4-7.8) | 6.2(5.4-8.7) | 6.6(5.4-7.5) | 0.893 |
| BMI(kg/m^2^) | 25.4(23.9-27.6) | 25.4(23.8-27.5) | 25.9(24.0-27.8) | 0.301 |
| Other variables |  |  |  |  |
| Urgent management of BP | 13(8.9) | 8(9.1) | 5(8.6) | 0.922 |
| Present as TIA | 26(18.3) | 13(14.8) | 13(22.4) | 0.238 |
| Referral | 36(24.7) | 22(25.0) | 14(24.1) | 0.906 |
| Transferring with EMS | 74(50.7) | 45(51.1) | 29(50.0) | 0.893 |
| Pre-notification | 17(11.6) | 17(19.3) | 0(0) | <0.001 |
| Lesion in AC | 115(78.8) | 67(76.1) | 48(82.8) | 0.403 |
| Multi-model imaging | 6(4.1) | 0(0) | 6(100.0) | 0.002 |
| Medical insurance | 95 (65.1) | 55(62.5) | 40(69.0) | 0.423 |
| Working days | 99(67.8) | 62(70.5) | 37(63.8) | 0.399 |
| Working hours | 77(52.7) | 50(56.8) | 27(46.6) | 0.224 |
