# Supplementary material for: Generalization of the Right Acute Stroke Prevention Strategies in Reducing in-Hospital Delays
Source: PLoS One. 2016 May 6;11(5):e0154972. doi: 10.1371/journal.pone.0154972 (PMC4859531; doi:10.1371/journal.pone.0154972)
Supplement: S2 Table — *In-hospital delay was defined as DTN time > 60 min. AC, anterior circulation; EMS, emergency medical service; NIHSS, National Institutes of Health Stroke Scale; TIA, transient ischemic attack. (DOC) [file pone.0154972.s003.doc]

| **S2 Table. Univariate Linear Regression Analysis to Identify Independent Variables that Affect In-hospital Delay of Post-intervention.** (In-hospital delay was defined as door-to-needle time ˃60min. NIHSS indicates National Institutes of Health Stroke Scale; AC, anterior circulation; TIA, transient ischemic attack; EMS, emergency medical service.)   | **Variables** | **Standardized coefficient** | ***P*** | | --- | --- | --- | | Onset-to-door time, min | -0.137 | 0.100 | | Door-to-evaluation time, min | 0.338 | <0.001 | | Door-to-imaging time, min | 0.250 | 0.002 | | Door-to-laboratory time, min | 0.409 | <0.001 | | Final-test-to-needle time, min | 0.535 | <0.001 | | Sex | 0.047 | 0.571 | | Age, year | 0.009 | 0.912 | | NIHSS | 0.008 | 0.925 | | Body mass index, kg/m^2^ | 0.065 | 0.438 | | Blood sugar, mmol/l | 0.037 | 0.655 | | Systolic blood pressure , mmHg | 0.027 | 0.742 | | Diastolic blood pressure, mmHg | -0.008 | 0.922 | | Urgent blood pressure control | -0.008 | 0.923 | | Lesion in the AC | 0.070 | 0.406 | | CT perfusion imaging | 0.255 | 0.002 | | Recent TIA | 0.098 | 0.241 | | Hypertension | -0.057 | 0.497 | | Diabetes | -0.096 | 0.250 | | Dyslipidemia | 0.090 | 0.281 | | Coronary heart disease | 0.025 | 0.769 | | Atrial fibrillation | 0.063 | 0.451 | | Prior stroke | 0.093 | 0.266 | | Smoking | 0.062 | 0.456 | | Heavy drink | 0.092 | 0.270 | | Admission date | -0.070 | 0.403 | | Working hour | -0.101 | 0.227 | | Medical insurance status | 0.066 | 0.426 | | Referral | -0.010 | 0.907 | | Transferring with EMS | -0.011 | 0.894 | | Pre-notification | -0.295 | <0.001 | |  |  |  | |
| --- | --- | --- | --- | --- | --- | --- | --- | --- | --- | --- | --- | --- | --- | --- | --- | --- | --- | --- | --- | --- | --- | --- | --- | --- | --- | --- | --- | --- | --- | --- | --- | --- | --- | --- | --- | --- | --- | --- | --- | --- | --- | --- | --- | --- | --- | --- | --- | --- | --- | --- | --- | --- | --- | --- | --- | --- | --- | --- | --- | --- | --- | --- | --- | --- | --- | --- | --- | --- | --- | --- | --- | --- | --- | --- | --- | --- | --- | --- | --- | --- | --- | --- | --- | --- | --- | --- | --- | --- | --- | --- | --- | --- | --- | --- | --- | --- |
